# Supplementary material for: How can new TB vaccines be effectively introduced in Indonesia? Insights from diverse stakeholders
Source: PLOS Glob Public Health. 2026 Jul 2;6(7):e0005537. doi: 10.1371/journal.pgph.0005537 (PMC13327203; doi:10.1371/journal.pgph.0005537)
Supplement: S1 Table — (DOCX) [file pgph.0005537.s001.docx]

**Supplementary File**

S1 Table. Participants and their represented institutions (with Participants’ Codes)

| **No** | **Stakeholders** | **Institutions** | **Participants’ codes** |
| --- | --- | --- | --- |
| 1 | Ministry of Health | National Tuberculosis Program (NTP) | GTB1 |
| 2 |  | Directorate of Immunization | GIM1 |
| 3 |  | Directorate of Immunization | GIM2 |
| 4 |  | Directorate General of Pharmacy and Medical Device | GFM1 |
| 5 |  | Expert Staff to Minister of Health for Health Financing | GPL4 |
| 6 |  | Health Development Policy Agency | GPL5 |
| 7 |  | Health Development Policy Agency | GPL6 |
| 8 | Ministry of National Development Planning | Ministry of National Development Planning | GPL1 |
| 9 | Indonesia Food and Drug Authority | Indonesian Food and Drug Authority | GPL2 |
| 10 |  | Indonesian Food and Drug Authority | GPL3 |
| 11 | Expert in Tuberculosis | Professor in pulmonology | ETB1 |
| 12 |  | Professor in internal medicine | ETB2 |
| 13 |  | Indonesian Paediatric Society | ETB3 |
| 14 |  | Indonesian Society of Respirology | ETB4 |
| 15 |  | Indonesian Society of Respirology | ETB9 |
| 16 |  | Indonesian Network of Tuberculosis Researchers | ETB5 |
| 17 |  | Expert Committee on Tuberculosis | ETB6 |
| 18 |  | Epidemiologist in tuberculosis | ETB7 |
| 19 |  | Epidemiologist in tuberculosis | ETB8 |
| 20 | Expert in Immunization | Indonesian Society of Internal Medicine | EIM1 |
| 21 |  | Indonesian Technical Advisory Group for Immunization | EIM2 |
| 22 | Expert in epidemiology | Epidemiologist and health policy expert | EPL1 |
| 23 | External, non-government organizations | WHO Indonesia Country Office | XIM1 |
| 24 |  | WHO Indonesia Country Office | XIM4 |
| 25 |  | UNICEF Indonesia Country Office | XIM2 |
| 26 |  | UNICEF Indonesia Country Office | XIM6 |
| 27 |  | Clinton Health Access Initiatives (CHAI) | XIM3 |
| 28 |  | Clinton Health Access Initiatives (CHAI) | XIM5 |
