# Supplementary material for: How can new TB vaccines be effectively introduced in Indonesia? Insights from diverse stakeholders
Source: PLOS Glob Public Health. 2026 Jul 2;6(7):e0005537. doi: 10.1371/journal.pgph.0005537 (PMC13327203; doi:10.1371/journal.pgph.0005537)
Supplement: Attachment — Submitted filename: Comments to Authors .docx [file pgph.0005537.s003.docx]

**Comments to Authors**

***General Comments***

This manuscript addresses a highly relevant and timely policy question: how Indonesia can prepare for the introduction of new TB vaccines. Given Indonesia’s substantial TB burden and its involvement in late-stage TB vaccine trials, the focus on stakeholder perspectives is both appropriate and valuable. The study brings together a diverse group of national and international stakeholders and provides practical insights that are directly relevant to policymakers and implementers.

The mixed-methods stakeholder consultation design is suitable for the study objectives, and the manuscript is generally well written, clearly structured, and transparent in reporting. The findings are coherent and thoughtfully discussed in relation to existing evidence and policy frameworks.

The manuscript would benefit from minor–moderate revisions aimed at strengthening methodological clarity, refining interpretation, and slightly tempering some conclusions to better reflect the consultative and exploratory nature of the data. These revisions would enhance transparency and further increase the manuscript’s value for decision-makers.

----------------------------

***Title***
The title is clear, accurate, and appropriately reflects the scope and focus of the study. No change required.

----------------------------

***Abstract***
The abstract is well structured and clearly summarizes the study objectives, methods, key findings, and conclusions.

**Minor comment:**
(Line 7): There is a minor grammatical error in the abstract (“introduction a new TB vaccine”); this should read “introduction **of** a new TB vaccine”

**Suggestions for improvement:**

1. Consider clarifying that the study is based on a stakeholder consultation rather than a representative survey.
2. In the Results section, it may be helpful to briefly acknowledge areas of divergence in stakeholder views (e.g., IGRA testing and rollout feasibility), to provide a more balanced summary.

----------------------------

***Introduction***

The introduction provides strong global and national context on TB burden and TB vaccine development and clearly establishes the policy relevance of the study.

**Minor comment:**

- (Line 67): define “UN High-Level Meeting” in full at first mention before using the abbreviation. This is also same for all other abbreviations such as BCG and so on.
- Line 69-72: consider rewording
- Line 73: I see a full stop in the middle. Delete it

**Suggestions for improvement:**

1. More explicitly articulate the specific gap this study addresses relative to previous stakeholder engagements and policy workshops.
2. Strengthen the rationale for conducting this consultation at the pre-licensure stage, highlighting the value of early implementation planning.

Minor editorial refinements would further improve clarity and flow.

----------------------------

***Methods***

The use of a mixed-methods stakeholder consultation is appropriate and well aligned with the research question.

**Suggestions for improvement:**

1. Briefly clarify how the quantitative and qualitative components were integrated analytically.
2. Acknowledge the potential influence of group dynamics during the consultation meeting and how this may have shaped responses.

**Participants and Sampling**

The inclusion of diverse stakeholders across policy, clinical, regulatory, and international organizations is a major strength.

**Suggestions for improvement:**

1. Provide slightly more detail on the criteria used to select participants within each stakeholder category.
2. Note that participants were purposively selected and that findings reflect expert perspectives rather than broader stakeholder or public views.
3. To enhance the transparency of the study and to inform future research, the authors are encouraged to share the English version of the questionnaire as supplementary material.

**Data Collection and Analysis**

The data collection process is clearly described, and the use of Slido® facilitated structured input from all participants.

**Suggestions for improvement:**

1. Expand the description of qualitative analysis to clarify whether coding was inductive, deductive, or combined.
2. Explicitly state that quantitative analyses were descriptive and exploratory.
3. For transparency, consider adding a brief description of the ranking and weighting approach used in Table 1 to the *Methods (Data Analysis)* section (e.g., how participant rankings were converted into weighted scores and summed). This would help readers unfamiliar with ranking exercises to better interpret the results.
4. The manuscript uses long dashes frequently and somewhat inconsistently. The authors may wish to review and standardize dash usage in line with journal style (e.g. lines 159&160).

----------------------------

***Results***

The results are clearly presented, and the combination of quantitative summaries with illustrative quotations enhances interpretability.

**Suggestions for improvement:**

1. Clearly distinguish descriptive findings from interpretive statements, particularly when discussing feasibility and prioritization.
2. Provide a brief explanation of the ranking and weighting approach used in Table 1 for readers unfamiliar with this method.
3. Emphasize that coverage estimates and timelines reflect stakeholder expectations rather than empirical projections.

----------------------------

***Discussion***

The discussion thoughtfully situates the findings within the broader TB vaccine and TB prevention literature and highlights important policy implications.

**Suggestions for improvement:**

1. Slightly temper interpretations where stakeholder agreement is discussed, clarifying that consensus reflects perceived priorities rather than confirmed feasibility.
2. When comparing TB vaccines to other vaccine programs (e.g., HPV), clarify differences in delivery context and public perception.
3. Frame regulatory and IGRA-related challenges as evolving considerations that may change as evidence and policies develop.

**Limitations**
The limitations section is appropriate and transparent.

**Suggestion:** Consider briefly acknowledging potential social desirability and group influence during the consultation.

----------------------------

***Conclusions***

The conclusions are consistent with the findings and appropriately emphasize the complexity of TB vaccine introduction.

**Suggestions for improvement:**

1. Slightly soften causal or definitive language to reflect the exploratory nature of the study.
2. Emphasize the role of this work in informing policy discussions rather than predicting policy outcomes.

----------------------------

***Tables, Figures, and Supplementary Material***

Tables and figures are clear and informative.

**Minor suggestion**: Expand figure captions slightly to improve standalone interpretability, particularly for Figure 1.

----------------------------

***Ethics, Data Availability***
Ethical approvals and data availability statements are appropriate.

----------------------------

***References***

References are current, relevant, and well selected.

**Minor suggestion:** Consider adding one reference on stakeholder consultation or participatory health policy research to further strengthen the methodological framing.

----------------------------

***Overall Recommendation***
This manuscript makes a valuable contribution to the literature on TB vaccine readiness and implementation planning in high-burden settings. With **minor–moderate revisions** to improve clarity, transparency, and interpretation, it would be well suited for publication in *PLOS Global Public Health*.
