# Supplementary material for: How can new TB vaccines be effectively introduced in Indonesia? Insights from diverse stakeholders
Source: PLOS Glob Public Health. 2026 Jul 2;6(7):e0005537. doi: 10.1371/journal.pgph.0005537 (PMC13327203; doi:10.1371/journal.pgph.0005537)
Supplement: Attachment — Submitted filename: Response to Reviewer TB vaccine Indonesia_v1.docx [file pgph.0005537.s005.docx]

Response to Reviewers

Dear Prof Suman Majumdar

Academic Editor
PLOS Global Public Health

We would like to express our sincere gratitude for the time and effort you have dedicated to reviewing our manuscript entitled *“How Can New TB Vaccines Be Effectively Introduced in Indonesia? Insights from Diverse Stakeholders.”* We greatly appreciate the constructive and insightful comments, which have helped us to substantially improve the clarity, rigor, and overall quality of the manuscript.

In response to your feedback, we have carefully revised the manuscript and addressed all comments point-by-point. We have also made editorial refinements throughout the text to enhance readability. All changes have been highlighted in the revised manuscript, and detailed responses to each comment are provided below.

**Editorial comments:**

1. Please include a complete copy of PLOS’ questionnaire on inclusivity in global research in your revised manuscript.

Response: The completed questionnaire is attached.

2. Please provide a detailed online Financial Disclosure statement and funder’s role.

Response: The statement is added (lines 526-528)

3. Please ensure that the funders and grant numbers match between the Financial Disclosure field and the Funding Information tab in your submission form. Note that the funders must be provided in the same order in both places as well.

Response: The numbers are matched.

4. Please update your online Competing Interests statement. If you have no competing interests to declare, please state: "The authors have declared that no competing interests exist."

Response: The statement is updated.

5. Please also provide non-author contact information (phone/email/hyperlink) for a data access committee, ethics committee, or other institutional body to which data requests may be sent.

Response: We added the non-author contact information in the online form.

*The Ethical Research Committee Faculty of Medicine Universitas Indonesia-Cipto Mangunkusumo Hospital*
Jl. Salemba 6, Jakarta Pusat
Telp. +62 021 315 7008
e-Mail: [ec_fkui@yahoo.com](mailto:ec_fkui@yahoo.com)

Website: [Contact | KOMITE ETIK PENELITIAN KESEHATAN](https://komite-etik.fk.ui.ac.id/p/index.php/p/about/contact)

6. Please provide separate main figure files in .tif or .eps format only and ensure that all files are under our size limit of 10MB.

Response: We replace that the main figure into .tif files

7. Please include a separate legend or caption for Figure 1 in your manuscript.

Response: The figure caption is included in the manuscript (line 224-226)

8. We have noticed that you have uploaded Supporting Information files, but you have not included a list of legends. Please add a full list of legends for your Supporting Information files before or after the references list.

Response: We added it accordingly.

9. If the reviewer comments include a recommendation to cite specific previously published works, please review and evaluate these publications to determine whether they are relevant and should be cited. There is no requirement to cite these works unless the editor has indicated otherwise.

Response: Thank you. We cited the works relevant to this study.

10. Please review your reference list to ensure that it is complete and correct.

Response: The reference list is complete and correct.

**Review #1 Comments.**

1. Overall, the article could do with an edit. E.g. IGRA is not spelled out in the abstract. Full stop in the middle of the sentence line 73. Line 74 - sentence needs to be checked. Line 173-174 summary and then summarized. Line 250 - needs sense checking.

Response:

- We spelled out IGRA in the abstract (line 46),
- remove some words (lines 74-75),
- revised the sentence (summary-summarised) to *“Key factors related to TB vaccine introduction is summarised in Figure 1.” (Lines 211-212)*, and
- revised the sentence to *“There were differing perspectives among participants regarding the level of coverage and the timeline for achieving it.” (lines 295-297)*

2. Participant section. Line 118 - you said 28 stakeholders were purposively selected for interview. I think this should read - for the consultation meeting not interview?

Response: Thank you for your review. Yes, it was for the consultation meeting. We revised accordingly.

3. Results section. Line 165 - should this read there were a total of 27 questionnaires completed?

Response: Yes, all completed the survey. We revised the sentence accordingly. (Lines 131-142)

4. N numbers are used inconsistently throughout. Would be helpful to standardize this across the sections.

Response: We revised them accordingly.

5. Line 210-211 - this seems more of a discussion section statement?

Response: There was one participant mentioning that issue including strategic advocacy to governing political parties. We revised the sentence to *“One participant cited that the issue of budget capacity could be overcome through strengthened political commitment, including strategic advocacy to governing political parties.” (lines 251-253)*

6. Discussion section. Lines 384 onwards don't really fit under the topic sentence for this paragraph and link more to the paragraph above on financing. Would be interesting to see the health systems and supply chain aspect this fleshed out a bit more as a paragraph in the discussion.

Response: Thank you for your review. We move the sentences into a new paragraph focusing on health system and supply chain as below.

*“From a health system perspective, the government needs to prepare the supply side to respond to potential increasing demand. This includes ensuring adequate cold chain capacity and integrating service delivery across relevant sectors. To deliver the vaccine effectively, a stepwise implementation approach, starting in a few provinces before scaling up nationwide, is preferred. It is also important to draw lessons from existing school-based vaccination programs, such as the school-based HPV vaccination programs, to optimize cost-efficiency[27–30]. The coordination between the Ministry of Health and the Ministry of Education has been well-established to support such program. However, while HPV vaccine is currently delivered primarily in elementary schools, a TB vaccine introduction targeting adolescents would extend to students in middle and high school. Given the distinct nature of TB transmission and varying risk perception across communities, a robust risk communication plan should be developed and strengthened. To support this implementation, the government should also develop sound financial planning and strengthen advocacy efforts for more affordable vaccine pricing.” (Lines 442-459)*

**Reviewer #2 Comments**

1. (Line 7): There is a minor grammatical error in the abstract (“introduction a new TB vaccine”); this should read “introduction of a new TB vaccine”

Response: We revised it accordingly.

2. Consider clarifying that the study is based on a stakeholder consultation rather than a representative survey.

Response: We agree with your suggestion. We would like to clarify that the study is based on stakeholder consultations, and we have ensured that the term “survey” is not used throughout the manuscript.

3. In the Results section, it may be helpful to briefly acknowledge areas of divergence in stakeholder views (e.g., IGRA testing and rollout feasibility), to provide a more balanced summary.

Response: Thank you for your suggestion. We revised the sentences to: *“Opinions diverged regarding the feasibility of vaccine rollout without Interferon-Gamma Release Assay (IGRA) testing: nine opposed vaccinating those with IGRA-negative, six abstained, and three argued that IGRA-testing was unnecessary and not efficient due to its high price.” (Lines 45-48)*

4. (Line 67): define “UN High-Level Meeting” in full at first mention before using the abbreviation. This is also same for all other abbreviations such as BCG and so on.

Response: We define all abbreviations in full as suggested.

5. Line 69-72: consider rewording

Response: We rephrased the sentence accordingly: *“Given that the current Bacillus Calmette–Guérin (BCG) vaccine is most effective at preventing severe disease in children[8], but its protection wanes over time, there remains no effective vaccines to protect adolescents and adults, two populations that contribute substantially to TB transmission.” (Lines 72-76)*

6. Line 73: I see a full stop in the middle. Delete it

Response: We deleted it.

7. More explicitly articulate the specific gap this study addresses relative to previous stakeholder engagements and policy workshops.

Response: We added a sentence to clarify the gap. *“However, there was limited detail regarding the specific strategies to be developed and the factors that should be considered from a broader, multi-perspective standpoint.” (Lines 99-101)*

8. Strengthen the rationale for conducting this consultation at the pre-licensure stage, highlighting the value of early implementation planning. Minor editorial refinements would further improve clarity and flow.

Response: We revised the rationale for conducting this study: *“To ensure effective implementation, particularly in Indonesia as a high TB burden country undergoing rapid programmatic developments, there is a need for strong evidence to inform early planning for TB vaccine introduction. Conducting a broader stakeholders consultative meeting at the pre-licensure stage is critical to proactively identify potential implementation strategies, anticipate health system constraints, and align policy and operational readiness prior to vaccine availability. This study therefore aimed to explore the perspectives of multiple stakeholders on the potential opportunities and challenges of introducing a new TB vaccine in Indonesia, taking into account relevant contextual, health system, and policy factors.” (Lines 105-117)*

9. Briefly clarify how the quantitative and qualitative components were integrated analytically.

Response: We clarified the description of analysis: *“Integration of quantitative and qualitative findings was conducted using a convergent mixed-methods approach, whereby findings from both components were compared, triangulated, and synthesized during the interpretation phase to generate comprehensive insights and provide a nuanced understanding of stakeholder views” (Lines 191-195)*

10. Acknowledge the potential influence of group dynamics during the consultation meeting and how this may have shaped responses.

Responses: We added a sentence to explicitly acknowledge the dynamics: *“Moderators facilitated individual reflection and group dialogue during the meeting to enrich the range of perspectives captured. Nevertheless, although participants answered independently, the potential influence of group dynamics on individual responses could not be entirely excluded.” (Lines 167-172)*

11. Provide slightly more detail on the criteria used to select participants within each stakeholder category.

Response: We added a sentence for clarity: *“All experts were required to have at least five years of experience in TB or vaccine research and practice, while all programmatic staff were required to have at least one year of experience in TB or vaccine programs.” (Lines 139-142)*

12. Note that participants were purposively selected and that findings reflect expert perspectives rather than broader stakeholder or public views.

Response: We added this in Limitation section: *“As participants were purposively selected and did not include perspectives from the general population, these findings reflect expert perspectives rather than public views.” (Lines 485-487)*

13. To enhance the transparency of the study and to inform future research, the authors are encouraged to share the English version of the questionnaire as supplementary material.

Response: We attached the English version of the questionnaire.

14. Expand the description of qualitative analysis to clarify whether coding was inductive, deductive, or combined.

Response: We added sentences to clarify our approaches: *“Two researchers (NDP and AF) independently reviewed and coded the responses using a primarily deductive approach, guided by the predefined themes and subthemes outlined in the questionnaire. At the same time, an inductive component was incorporated to allow for the identification of emerging themes not captured in the initial framework.” (Lines 186-190)*

15. Explicitly state that quantitative analyses were descriptive and exploratory.

Response: We revised and explicitly state the approach: *“Quantitative data from the closed-ended questionnaire items were analysed descriptively and exploratory; and they were displayed as frequencies and proportions.” (Line 178-180)*

16. For transparency, consider adding a brief description of the ranking and weighting approach used in Table 1 to the Methods (Data Analysis) section (e.g., how participant rankings were converted into weighted scores and summed). This would help readers unfamiliar with ranking exercises to better interpret the results.

Response: We added the explanation in Methods section. *“Regarding the target population, participants were asked to rank target populations from the highest to lowest priority. Rankings were converted into weighted scores by assigning higher weights to higher-priority ranks (e.g., rank 1=6 points to rank 6= point), and the weighted scores were summed across participants for each population.” (Lines 180-184)*

17. The manuscript uses long dashes frequently and somewhat inconsistently. The authors may wish to review and standardize dash usage in line with journal style (e.g. lines 159&160).

Response: Thank you for your detailed observations. We have reduced the use of long dashes, removed them where appropriate, and expanded the text to improve clarity.

18. Clearly distinguish descriptive findings from interpretive statements, particularly when discussing feasibility and prioritization.

Response: They are descriptive findings. We added two sentences to clarify this. *“We also assessed feasibility based on participants’ perspectives on whether the target populations had been successfully reached in previous programs. Most participants perceived that all populations would be feasible to reach, except for people with diabetes.” (Lines 272-275)*

19. Provide a brief explanation of the ranking and weighting approach used in Table 1 for readers unfamiliar with this method.

Response: We added the explanation in the Methods section.

20. Emphasize that coverage estimates and timelines reflect stakeholder expectations rather than empirical projections.

Response: We added “perceived” in the sentence. (Line 275).

21. Slightly temper interpretations where stakeholder agreement is discussed, clarifying that consensus reflects perceived priorities rather than confirmed feasibility.

Response: We added in the first paragraph of Discussion section: *“Although the insights gained from this study reflects more perceived priorities and not necessarily confirmed feasibility, the findings underscore the multifaceted nature of TB vaccine introduction and the need for a comprehensive, coordinated approach to ensure its successful and sustainable implementation.” (Lines 384-387)*

22. When comparing TB vaccines to other vaccine programs (e.g., HPV), clarify differences in delivery context and public perception.

Response: We added a sentence in Discussions section: *“*It is also important to draw lessons from existing school-based vaccination programs, such as the school-based HPV vaccination program, to optimize cost-efficiency[27–30]. The coordination between the Ministry of Health and the Ministry of Education has been well-established to support such program. However, while HPV vaccine is currently delivered primarily in elementary schools, a TB vaccine introduction targeting adolescents would extend to students in middle and high school. Given the distinct nature of TB transmission and varying risk perception across communities, a robust risk communication plan should be developed and strengthened*.” (Lines 445-453)*

23. Frame regulatory and IGRA-related challenges as evolving considerations that may change as evidence and policies develop.

Response: We added: *“However, the evidence regarding IGRA testing may evolve by the time TB vaccines are ready to be deployed, and regulations may also change. Despite these potential developments in evidence and policy, these considerations highlight the importance of designing vaccines and delivery strategies that are operationally feasible and financially sustainable in real-world contexts.*” (Lines 465-469)

24. Suggestion: Consider briefly acknowledging potential social desirability and group influence during the consultation.

Response: We acknowledged the potential desirability bias and group dynamics in the Methods section, “*Since participants completed the questionnaire independently, the potential for social desirability bias was reduced. Moderators facilitated individual reflection and group dialogue during the meeting to enrich the range of perspectives captured. Nevertheless, although participants answered independently, the potential influence of group dynamics on individual responses could not be entirely excluded.*” (Lines 167-172).

25. Slightly soften causal or definitive language to reflect the exploratory nature of the study.

Response: We reduced the use of definitive language such as “affect,” “influence,” or “determine” throughout the manuscript to reflect the exploratory nature of the findings.

26. Emphasize the role of this work in informing policy discussions rather than predicting policy outcomes.

Response: Thank you for your suggestions. We added sentences in the Discussion: *“The discussion dynamics and findings are useful for informing further policy discussions, although they do not constitute final policy recommendations for TB vaccine delivery. We acknowledge that policy directions may evolve over time as TB vaccines become available; however, the underlying considerations and guiding principles are likely to remain consistent.” (Lines 476-480)*

27. Minor suggestion: Expand figure captions slightly to improve standalone interpretability, particularly for Figure 1.

Response: We revised the caption to *“Figure 1. Key factors for TB vaccine introduction. Circle size represents the relative importance or priority of each factor as perceived by participants.”*

28. Minor suggestion: Consider adding one reference on stakeholder consultation or participatory health policy research to further strengthen the methodological framing.

Response: We added one reference regarding consultation meeting, *“Petkovic J, Magwood O, Lytvyn L, Khabsa J, Concannon TW, Welch V, et al. Key issues for stakeholder engagement in the development of health and healthcare guidelines. Res Involv Engagem. 2023;9:27. https://doi.org/10.1186/s40900-023-00433-6.”*

**Reviewer #3 Comments**

1. While the submission is strong, I recommend a minor revision to address a specific gap in the discussion. Specifically, the authors conclude that local manufacturing is not a prerequisite for vaccine introduction. To better justify this finding, the authors should provide a comparative analysis between local and non-local manufacturing models.

Response: We added a paragraph regarding the local manufacturing. *“Local manufacturing was identified as one of factors to consider, although it was not viewed as a high priority. The vaccine is currently being developed and tested by global manufacturers, making local replication or independent development challenging in the short term. However, when preparing the TB vaccine introduction, advocacy efforts should emphasize the importance of technology transfer to enable future local production. This would support long-term sustainability, improve affordability through reduced costs, and contribute to strengthening local technological capacity.”* (Lines 413-420)

2. Additionally, I request that the authors provide more structured outline or table categorizing the introduction of the TB vaccine across Availability, Acceptability, Affordability, and Price framework. Addressing these points will significantly strengthen the practical implications of the study.

Response: This study focused on exploring factors related to TB vaccine introduction. We acknowledge that certain aspects could not be fully assessed at this stage, including availability and acceptability, as the vaccine is not yet available. Regarding affordability and pricing, the exact vaccine price remains unknown. However, we addressed this issue in the Discussion section: *“Participants expressed concerns about vaccine pricing and advocated for it to be ‘as low as possible’, noting that the government would need to secure long-term funding. Price negotiation was considered critical to maximize population coverage and ensure broad public health impact” (Lines 423–425).*

We hope that the revisions have adequately addressed all concerns and that the manuscript is now suitable for publication in *PLOS Global Public Health*. We believe that this study provides timely and policy-relevant insights into the early planning and implementation considerations for TB vaccine introduction in Indonesia.

Thank you again for your thoughtful and constructive feedback. We look forward to your further consideration.

Sincerely,
Ahmad Fuady, *on behalf of all authors*
