# Supplementary material for: How can new TB vaccines be effectively introduced in Indonesia? Insights from diverse stakeholders
Source: PLOS Glob Public Health. 2026 Jul 2;6(7):e0005537. doi: 10.1371/journal.pgph.0005537 (PMC13327203; doi:10.1371/journal.pgph.0005537)
Supplement: Peer review history

# Peer review: Decision Letter 0

**DOI:** 10.1371/journal.pgph.0005537.r001

**Authors:** Suman Majumdar

3 Mar 2026

PGPH-D-25-03400

How Can New TB Vaccines Be Effectively Introduced in Indonesia? Insights from Diverse Stakeholders

PLOS Global Public Health

Dear Dr. Fuady,

Thank you for submitting your manuscript to PLOS Global Public Health. After careful consideration, we feel that it has merit but does not fully meet PLOS Global Public Health’s publication criteria as it currently stands. Therefore, we invite you to submit a revised version of the manuscript that addresses the points raised during the review process.

Please submit your revised manuscript by Apr 02 2026 11:59PM. If you will need more time than this to complete your revisions, please reply to this message or contact the journal office at globalpubhealth@plos.org. When you're ready to submit your revision, log on to https://www.editorialmanager.com/pgph/ and select the 'Submissions Needing Revision' folder to locate your manuscript file.

Please include the following items when submitting your revised manuscript:

- A letter that responds to each point raised by the editor and reviewer(s). You should upload this letter as a separate file labeled 'Response to Reviewers'.
- A marked-up copy of your manuscript that highlights changes made to the original version. You should upload this as a separate file labeled 'Revised Manuscript with Track Changes'.
- An unmarked version of your revised paper without tracked changes. You should upload this as a separate file labeled 'Manuscript'.

Guidelines for resubmitting your figure files are available below the reviewer comments at the end of this letter.

We look forward to receiving your revised manuscript.

Kind regards,

A/Prof Suman Majumdar

Academic Editor

PLOS Global Public Health

Journal Requirements:

1. Please include a complete copy of PLOS’ questionnaire on inclusivity in global research in your revised manuscript. Our policy for research in this area aims to improve transparency in the reporting of research performed outside of researchers’ own country or community. The policy applies to researchers who have travelled to a different country to conduct research, research with Indigenous populations or their lands, and research on cultural artefacts. The questionnaire can also be requested at the journal’s discretion for any other submissions, even if these conditions are not met. Please find more information on the policy and a link to download a blank copy of the questionnaire here: https://journals.plos.org/globalpublichealth/s/best-practices-in-research-reporting. Please upload a completed version of your questionnaire as Supporting Information when you resubmit your manuscript.

2. Please provide a detailed online Financial Disclosure statement. This is published with the article. It must therefore be completed in full sentences and contain the exact wording you wish to be published.

a) State the initials, alongside each funding source, of each author to receive each grant. For example: “This work was supported by the National Institutes of Health (####### to AM; ###### to CJ) and the National Science Foundation (###### to AM).”

b) State what role the funders took in the study. If the funders had no role in your study, please state: “The funders had no role in study design, data collection and analysis, decision to publish, or preparation of the manuscript.”

For more information, please go to our submission guidelines:

https://journals.plos.org/globalpublichealth/s/submission-guidelines#loc-financial-disclosure-statement

3. Please ensure that the funders and grant numbers match between the Financial Disclosure field and the Funding Information tab in your submission form. Note that the funders must be provided in the same order in both places as well.

4. Please update your online Competing Interests statement. If you have no competing interests to declare, please state: "The authors have declared that no competing interests exist."

5. In this instance it seems there may be acceptable restrictions in place that prevent the public sharing of your minimal data. However, in line with our goal of ensuring long-term data availability to all interested researchers, PLOS’ Data Policy states that authors cannot be the sole named individuals responsible for ensuring data access (http://journals.plos.org/globalpublichealth/s/data-availability#loc-acceptable-data-sharing-methods).

Data requests to a non-author institutional point of contact, such as a data access or ethics committee, helps guarantee long term stability and availability of data. Providing interested researchers with a durable point of contact ensures data will be accessible even if an author changes email addresses, institutions, or becomes unavailable to answer requests.

Before we proceed with your manuscript, please also provide non-author contact information (phone/email/hyperlink) for a data access committee, ethics committee, or other institutional body to which data requests may be sent. If no institutional body is available to respond to requests for your minimal data, please consider if there any institutional representatives who did not collaborate in the study, and are not listed as authors on the manuscript, who would be able to hold the data and respond to external requests for data access? If so, please provide their contact information (i.e., email address). Please also provide details on how you will ensure persistent or long-term data storage and availability.

6. Please provide separate main figure files in .tif or .eps format only and ensure that all files are under our size limit of 10MB.

For more information about how to convert your figure files please see our guidelines: https://journals.plos.org/globalpublichealth/s/figures

7. Please include a separate legend or caption for Figure 1 in your manuscript.

8. We have noticed that you have uploaded Supporting Information files, but you have not included a list of legends. Please add a full list of legends for your Supporting Information files before or after the references list.

9. If the reviewer comments include a recommendation to cite specific previously published works, please review and evaluate these publications to determine whether they are relevant and should be cited. There is no requirement to cite these works unless the editor has indicated otherwise.

10. Please review your reference list to ensure that it is complete and correct. If you have cited papers that have been retracted, please include the rationale for doing so in the manuscript text, or remove these references and replace them with relevant current references. Any changes to the reference list should be mentioned in the rebuttal letter that accompanies your revised manuscript. If you need to cite a retracted article, indicate the article’s retracted status in the References list and also include a citation and full reference for the retraction notice.

[Note: HTML markup is below. Please do not edit.]

Reviewers' comments:

Reviewer's Responses to Questions

**Comments to the Author**

1. Does this manuscript meet PLOS Global Public Health’s publication criteria? Is the manuscript technically sound, and do the data support the conclusions? The manuscript must describe methodologically and ethically rigorous research with conclusions that are appropriately drawn based on the data presented.

Reviewer #1: Yes

Reviewer #2: Yes

Reviewer #3: Yes

**********

2. Has the statistical analysis been performed appropriately and rigorously?

Reviewer #1: Yes

Reviewer #2: Yes

Reviewer #3: Yes

**********

3. Have the authors made all data underlying the findings in their manuscript fully available (please refer to the Data Availability Statement at the start of the manuscript PDF file)?

The PLOS Data policy requires authors to make all data underlying the findings described in their manuscript fully available without restriction, with rare exception. The data should be provided as part of the manuscript or its supporting information, or deposited to a public repository. For example, in addition to summary statistics, the data points behind means, medians and variance measures should be available. If there are restrictions on publicly sharing data—e.g. participant privacy or use of data from a third party—those must be specified.

Reviewer #1: Yes

Reviewer #2: Yes

Reviewer #3: Yes

**********

4. Is the manuscript presented in an intelligible fashion and written in standard English?

PLOS Global Public Health does not copyedit accepted manuscripts, so the language in submitted articles must be clear, correct, and unambiguous. Any typographical or grammatical errors should be corrected at revision, so please note any specific errors here.

Reviewer #1: Yes

Reviewer #2: No

Reviewer #3: Yes

**********

5. Review Comments to the Author

Please use the space provided to explain your answers to the questions above. You may also include additional comments for the author, including concerns about dual publication, research ethics, or publication ethics. (Please upload your review as an attachment if it exceeds 20,000 characters)

Reviewer #1: This is an important paper to highlight the need for countries to start preparing for the potential roll-out of an adult TB vaccine from 2030, with interesting results from stakeholder engagement in Indonesia.

Overall, the article could do with an edit. E.g. IGRA is not spelled out in the abstract. Full stop in the middle of the sentence line 73. Line 74 - sentence needs to be checked. Line 173-174 summary and then summarized. Line 250 - needs sense checking.

Participant section

Line 118 - you said 28 stakeholders were purposively selected for interview. I think this should read - for the consultation meeting not interview?

Results section

Line 165 - should this read there were a total of 27 questionnaires completed?

N numbers are used inconsistently throughout. Would be helpful to standardize this across the sections.

Line 210-211 - this seems more of a discussion section statement?

Discussion section

Lines 384 onwards don't really fit under the topic sentence for this paragraph and link more to the paragraph above on financing. Would be interesting to see the health systems and supply chain aspect this fleshed out a bit more as a paragraph in the discussion.

Reviewer #2: Comments to Authors

General Comments

This manuscript addresses a highly relevant and timely policy question: how Indonesia can prepare for the introduction of new TB vaccines. Given Indonesia’s substantial TB burden and its involvement in late-stage TB vaccine trials, the focus on stakeholder perspectives is both appropriate and valuable. The study brings together a diverse group of national and international stakeholders and provides practical insights that are directly relevant to policymakers and implementers.

The mixed-methods stakeholder consultation design is suitable for the study objectives, and the manuscript is generally well written, clearly structured, and transparent in reporting. The findings are coherent and thoughtfully discussed in relation to existing evidence and policy frameworks.

The manuscript would benefit from minor–moderate revisions aimed at strengthening methodological clarity, refining interpretation, and slightly tempering some conclusions to better reflect the consultative and exploratory nature of the data. These revisions would enhance transparency and further increase the manuscript’s value for decision-makers.

----------------------------

Title

The title is clear, accurate, and appropriately reflects the scope and focus of the study. No change required.

----------------------------

Abstract

The abstract is well structured and clearly summarizes the study objectives, methods, key findings, and conclusions.

Minor comment:

(Line 7): There is a minor grammatical error in the abstract (“introduction a new TB vaccine”); this should read “introduction of a new TB vaccine”

Suggestions for improvement:

1. Consider clarifying that the study is based on a stakeholder consultation rather than a representative survey.

2. In the Results section, it may be helpful to briefly acknowledge areas of divergence in stakeholder views (e.g., IGRA testing and rollout feasibility), to provide a more balanced summary.

----------------------------

Introduction

The introduction provides strong global and national context on TB burden and TB vaccine development and clearly establishes the policy relevance of the study.

Minor comment:

• (Line 67): define “UN High-Level Meeting” in full at first mention before using the abbreviation. This is also same for all other abbreviations such as BCG and so on.

• Line 69-72: consider rewording

• Line 73: I see a full stop in the middle. Delete it

Suggestions for improvement:

1. More explicitly articulate the specific gap this study addresses relative to previous stakeholder engagements and policy workshops.

2. Strengthen the rationale for conducting this consultation at the pre-licensure stage, highlighting the value of early implementation planning.

Minor editorial refinements would further improve clarity and flow.

----------------------------

Methods

The use of a mixed-methods stakeholder consultation is appropriate and well aligned with the research question.

Suggestions for improvement:

1. Briefly clarify how the quantitative and qualitative components were integrated analytically.

2. Acknowledge the potential influence of group dynamics during the consultation meeting and how this may have shaped responses.

Participants and Sampling

The inclusion of diverse stakeholders across policy, clinical, regulatory, and international organizations is a major strength.

Suggestions for improvement:

1. Provide slightly more detail on the criteria used to select participants within each stakeholder category.

2. Note that participants were purposively selected and that findings reflect expert perspectives rather than broader stakeholder or public views.

3. To enhance the transparency of the study and to inform future research, the authors are encouraged to share the English version of the questionnaire as supplementary material.

Data Collection and Analysis

The data collection process is clearly described, and the use of Slido® facilitated structured input from all participants.

Suggestions for improvement:

1. Expand the description of qualitative analysis to clarify whether coding was inductive, deductive, or combined.

2. Explicitly state that quantitative analyses were descriptive and exploratory.

3. For transparency, consider adding a brief description of the ranking and weighting approach used in Table 1 to the Methods (Data Analysis) section (e.g., how participant rankings were converted into weighted scores and summed). This would help readers unfamiliar with ranking exercises to better interpret the results.

4. The manuscript uses long dashes frequently and somewhat inconsistently. The authors may wish to review and standardize dash usage in line with journal style (e.g. lines 159&160).

----------------------------

Results

The results are clearly presented, and the combination of quantitative summaries with illustrative quotations enhances interpretability.

Suggestions for improvement:

1. Clearly distinguish descriptive findings from interpretive statements, particularly when discussing feasibility and prioritization.

2. Provide a brief explanation of the ranking and weighting approach used in Table 1 for readers unfamiliar with this method.

3. Emphasize that coverage estimates and timelines reflect stakeholder expectations rather than empirical projections.

----------------------------

Discussion

The discussion thoughtfully situates the findings within the broader TB vaccine and TB prevention literature and highlights important policy implications.

Suggestions for improvement:

1. Slightly temper interpretations where stakeholder agreement is discussed, clarifying that consensus reflects perceived priorities rather than confirmed feasibility.

2. When comparing TB vaccines to other vaccine programs (e.g., HPV), clarify differences in delivery context and public perception.

3. Frame regulatory and IGRA-related challenges as evolving considerations that may change as evidence and policies develop.

Limitations

The limitations section is appropriate and transparent.

Suggestion: Consider briefly acknowledging potential social desirability and group influence during the consultation.

----------------------------

Conclusions

The conclusions are consistent with the findings and appropriately emphasize the complexity of TB vaccine introduction.

Suggestions for improvement:

1. Slightly soften causal or definitive language to reflect the exploratory nature of the study.

2. Emphasize the role of this work in informing policy discussions rather than predicting policy outcomes.

----------------------------

Tables, Figures, and Supplementary Material

Tables and figures are clear and informative.

Minor suggestion: Expand figure captions slightly to improve standalone interpretability, particularly for Figure 1.

----------------------------

Ethics, Data Availability

Ethical approvals and data availability statements are appropriate.

----------------------------

References

References are current, relevant, and well selected.

Minor suggestion: Consider adding one reference on stakeholder consultation or participatory health policy research to further strengthen the methodological framing.

----------------------------

Reviewer #3: Overall, the study employs a rigorous and relevant qualitative methodology, and the manuscript is written with a high standard of clarity.

While the submission is strong, I recommend a minor revision to address a specific gap in the discussion. Specifically, the authors conclude that local manufacturing is not a prerequisite for vaccine introduction. To better justify this finding, the authors should provide a comparative analysis between local and non-local manufacturing models.

Additionally, I request that the authors provide more structured outline or table categorizing the introduction of the TB vaccine across Availability, Acceptability, Affordability, and Price framework. Addressing these points will significantly strengthen the practical implications of the study.

**********

6. PLOS authors have the option to publish the peer review history of their article (what does this mean?). If published, this will include your full peer review and any attached files.

**Do you want your identity to be public for this peer review?**  If you choose “no”, your identity will remain anonymous but your review may still be made public.

For information about this choice, including consent withdrawal, please see our Privacy Policy.

Reviewer #1: No

Reviewer #2: **Yes:** Mohammed Alfaqeeh

Reviewer #3: No

**********

[NOTE: If reviewer comments were submitted as an attachment file, they will be attached to this email and accessible via the submission site. Please log into your account, locate the manuscript record, and check for the action link "View Attachments". If this link does not appear, there are no attachment files.]

Figure Resubmissions:

While revising your submission, we strongly recommend that you use PLOS’s NAAS tool (https://ngplosjournals.pagemajik.ai/artanalysis) to test your figure files. NAAS can convert your figure files to the TIFF file type and meet basic requirements (such as print size, resolution), or provide you with a report on issues that do not meet our requirements and that NAAS cannot fix.

After uploading your figures to PLOS’s NAAS tool - https://ngplosjournals.pagemajik.ai/artanalysis, NAAS will process the files provided and display the results in the "Uploaded Files" section of the page as the processing is complete. If the uploaded figures meet our requirements (or NAAS is able to fix the files to meet our requirements), the figure will be marked as "fixed" above. If NAAS is unable to fix the files, a red "failed" label will appear above. When NAAS has confirmed that the figure files meet our requirements, please download the file via the download option, and include these NAAS processed figure files when submitting your revised manuscript.

Attachment Submitted filename: Comments to Authors .docx

# Author comment: Author response to Decision Letter 1

**DOI:** 10.1371/journal.pgph.0005537.r002

14 Apr 2026

Attachment Submitted filename: Response to Reviewer TB vaccine Indonesia_v1.docx

# Peer review: Decision Letter 1

**DOI:** 10.1371/journal.pgph.0005537.r003

**Authors:** Suman Majumdar, Suman Majumdar

10 Jun 2026

How Can New TB Vaccines Be Effectively Introduced in Indonesia? Insights from Diverse Stakeholders

PGPH-D-25-03400R1

Dear Dr Fuady,

We are pleased to inform you that your manuscript 'How Can New TB Vaccines Be Effectively Introduced in Indonesia? Insights from Diverse Stakeholders' has been provisionally accepted for publication in PLOS Global Public Health.

We note that reviewer 2 has made an additional suggestion to cite their work. Since this is revision is not necessary, I have issued an accept decision.

Before your manuscript can be formally accepted you will need to complete some formatting changes, which you will receive in a follow up email. A member of our team will be in touch with a set of requests.

Please note that your manuscript will not be scheduled for publication until you have made the required changes, so a swift response is appreciated.

IMPORTANT: The editorial review process is now complete. PLOS will only permit corrections to spelling, formatting or significant scientific errors from this point onwards. Requests for major changes, or any which affect the scientific understanding of your work, will cause delays to the publication date of your manuscript.

If your institution or institutions have a press office, please notify them about your upcoming paper to help maximize its impact. If they'll be preparing press materials, please inform our press team as soon as possible -- no later than 48 hours after receiving the formal acceptance. Your manuscript will remain under strict press embargo until 2 pm Eastern Time on the date of publication. For more information, please contact globalpubhealth@plos.org.

Thank you again for supporting Open Access publishing; we are looking forward to publishing your work in PLOS Global Public Health.

Best regards,

Katherine Kokkinias, Ph.D.

Staff Editor, PLOS Global Public Health

on behalf of

Suman Majumdar

Academic Editor

PLOS Global Public Health

***********************************************************

Reviewer Comments (if any, and for reference):

Reviewer's Responses to Questions

**Comments to the Author**

1. If the authors have adequately addressed your comments raised in a previous round of review and you feel that this manuscript is now acceptable for publication, you may indicate that here to bypass the “Comments to the Author” section, enter your conflict of interest statement in the “Confidential to Editor” section, and submit your "Accept" recommendation.

Reviewer #1: All comments have been addressed

Reviewer #2: All comments have been addressed

Reviewer #3: All comments have been addressed

**********

2. Does this manuscript meet PLOS Global Public Health’s publication criteria? Is the manuscript technically sound, and do the data support the conclusions? The manuscript must describe methodologically and ethically rigorous research with conclusions that are appropriately drawn based on the data presented.

Reviewer #1: Yes

Reviewer #2: Yes

Reviewer #3: Yes

**********

3. Has the statistical analysis been performed appropriately and rigorously?

Reviewer #1: Yes

Reviewer #2: Yes

Reviewer #3: Yes

**********

4. Have the authors made all data underlying the findings in their manuscript fully available (please refer to the Data Availability Statement at the start of the manuscript PDF file)?

The PLOS Data policy requires authors to make all data underlying the findings described in their manuscript fully available without restriction, with rare exception. The data should be provided as part of the manuscript or its supporting information, or deposited to a public repository. For example, in addition to summary statistics, the data points behind means, medians and variance measures should be available. If there are restrictions on publicly sharing data—e.g. participant privacy or use of data from a third party—those must be specified.

Reviewer #1: Yes

Reviewer #2: Yes

Reviewer #3: Yes

**********

5. Is the manuscript presented in an intelligible fashion and written in standard English?

PLOS Global Public Health does not copyedit accepted manuscripts, so the language in submitted articles must be clear, correct, and unambiguous. Any typographical or grammatical errors should be corrected at revision, so please note any specific errors here.

Reviewer #1: Yes

Reviewer #2: Yes

Reviewer #3: Yes

**********

6. Review Comments to the Author

Please use the space provided to explain your answers to the questions above. You may also include additional comments for the author, including concerns about dual publication, research ethics, or publication ethics. (Please upload your review as an attachment if it exceeds 20,000 characters)

Reviewer #1: Thank you for responding and addressing the reviewer comments. No further comments.

Reviewer #2: I appreciate the authors’ efforts in revising the manuscript. The paper has improved significantly, and the revisions have addressed the previous comments in a satisfactory manner.

One additional suggestion relates to the Discussion section. While the manuscript clearly presents findings from the stakeholder consultation, it would benefit from placing greater emphasis on the importance and added value of this approach in informing policy and decision-making in Indonesia. In particular, the authors could elaborate on how stakeholder consultation contributes to evidence-informed priority setting by integrating technical considerations (e.g., effectiveness, cost) with value-based aspects (e.g., acceptability, equity), and how this process supports decision-makers in planning the introduction of new TB vaccines. Strengthening this aspect would enhance the policy relevance of the study and better highlight its contribution beyond descriptive findings. The authors may consider citing the following reference to support this discussion: https://doi.org/10.1186/s12939-025-02668-z

Reviewer #3: (No Response)

**********

7. PLOS authors have the option to publish the peer review history of their article (what does this mean?). If published, this will include your full peer review and any attached files.

**Do you want your identity to be public for this peer review?**  If you choose “no”, your identity will remain anonymous but your review may still be made public.

For information about this choice, including consent withdrawal, please see our Privacy Policy.

Reviewer #1: No

Reviewer #2: **Yes:** Mohammed Alfaqeeh

Reviewer #3: **Yes:** Erlina Burhan

**********
